# Data to establish the optimal standard regimen and predicting the response to docetaxel therapy

**DOI:** 10.1016/j.dib.2015.09.033

**Published:** 2015-10-09

**Authors:** Emad Y. Moawad

**Affiliations:** Department of Engineering, Ain Shams University, 217 Alhegaz Street, Alnozha, 11351 Cairo, Egypt

## Abstract

This paper contains data to establish the optimal standard regimen and predicting the response to docetaxel therapy (Moawad, 2014) [1]. Docetaxel has been in use for over a decade without demonstrating data indicates a predictable response in the treatment of cancer. Data of puzzling response to docetaxel therapy was due to its cell cycle specific effect. Although several administered schedules were investigated, the relative therapeutic advantage of high versus low doses has not been identified yet. Also the antitumor target of docetaxel has not yet been identified to optimize therapy by predicting the response of patients prior to therapy to provide a protection against treatment failure. In the present paper, we demonstrate the data used to optimize docetaxel therapy and investigate the possibility of predicting for the first time the antitumor target of docetaxel.

## 1 Specifications Table

**Table:** 

Subject area	Medical oncology
More specific subject area	Docetaxel therapy – cancer staging – identifying the effectiveness of antitumor drugs.
Type of data	Table, text file, graph.
How data was acquired	Data was acquired from each of a previously published data of docetaxel cancer growth inhibition in vivo and data of previously published methods for cancer staging and identifying effectiveness of antitumor drugs.
Data format	Dose modeling was performed by analyzing the acquired data.
Experimental factors	Staging and grading tumors.
Experimental features	Tumor growth inhibition in vivo*.*
Data source location	Earlier studies conducted by the author of current article – earlier studies conducted by different schools of medicine published on the Internet.
Data accessibility	Staging and grading cancer.
[http://www.ncbi.nlm.nih.gov/pubmed/25013524
http://www.ncbi.nlm.nih.gov/pubmed/26069487
http://www.ncbi.nlm.nih.gov/pubmed/26069495
http://link.springer.com/article/10.1007/s00580-012-1603-6
http://www.hrpub.org/journals/article_info.php?aid=660]
[Identifying effectiveness of antitumor drugs.
http://www.ncbi.nlm.nih.gov/pubmed/24248635
http://www.ncbi.nlm.nih.gov/pubmed/25985771
http://www.ncbi.nlm.nih.gov/pubmed/26346504
http://www.ncbi.nlm.nih.gov/pubmed/25298625
http://link.springer.com/article/10.1007/s40944-015-0001-9]
Docetaxel cancer growth inhibition.
[http://cancerres.aacrjournals.org/content/67/1/281
http://onlinelibrary.wiley.com/doi/10.1002/1097-0045(20000901)44:4%3C275::AID-PROS3%3E3.0.CO;2-9/pdf
http://www.sciencedirect.com/science/article/pii/S0014299908003683
http://cancerres.aacrjournals.org/content/66/9/4816
http://cancerres.aacrjournals.org/content/67/8/3818
http://mct.aacrjournals.org/content/4/6/1004
http://clincancerres.aacrjournals.org/content/15/2/543
http://archotol.jamanetwork.com/article.aspx?articleid=649061
http://www.ncbi.nlm.nih.gov/pubmed/25771878]

## 2 Value of the data

● Although docetaxel has been in use for over a decade, optimal dosing and scheduling are still the most important issues regarding the use of docetaxel [2–9].
● In the same time, predicting patient׳s response has become a necessity to preserve patient׳s right against treatment failure or non-optimal treatments [10–17].
● The acquired data was for identifying the energy yield by docetaxel doses to investigate the possibility of predicting for the first time the antitumor target of docetaxel.
● Assessment of the efficient regimen for optimizing cell-cycle specific therapy would be based on achieving an accumulated doubling time–energy conversion in the tumor cells by the regimen doses [1,12].
● The higher the energy yields by the same docetaxel dose the more effectiveness of the applied regimen and vice versa.
● Then, efficiency of those applied standard and metronomic regimens on different types of tumor models would be determined to assess the specifications of the personalized treatment schedule [10–14].
● The correlation and regression between the energy yield by the applied docetaxel doses in optimal schedules (dependent variable) and value of those doses (independent variable) would be investigated. If both variables were perfectly correlated, the target of our thesis would be achieved.
● In such a case, a dose–energy model with perfect fit for docetaxel would be constructed to administer the optimal (personalized) dose in an efficient schedule as conducted before in earlier studies [10–14].
● Accordingly, the therapeutic response of cancer to docetaxel could be predicted prior to therapy by identifying each of patient׳s histologic grade (H_G Control_)—in vitro or in vivo—and energy yield by the proposed dose using the constructed dose–energy model of docetaxel [10–20].

## 3 Data

### 3.1 Docetaxel cancer growth inhibition

Data shows that schedule of the applied regimen is responsible for the puzzling response to therapy due to the cell cycle specific effect of docetaxel.

### 3.2 Clinical model of staging and grading cancer

The processes of tumor formation and cancer therapy are based mainly on the concept of doubling time–energy conversion (DT–EC) in which the conversion of doubling time into growth energy takes place [10–21]. The fundamental principle for cell cycle duration in relation to the physical energy condition of a cell has been derived and confirmed [17,21]. In which, the duration of the mitosis stage is defined by cell doubling time or division time and denoted by $t_{D}$. While the growth energy $\left(E_{G}\right)$ of the biological cell in terms of $t_{D}$ was expressed by the DT–EC formula:

(1) $$E_{G}=\ln{\left(\ln\left(\frac{\mathrm{ln2}}{t_{D}}\right)\right)}^{2}\mathrm{Emad}$$

which is known also by Emad formula referring to the unit used in identifying the converted energy [17–26]. The Emad unit of each of the biological cell growth energy and the radionuclide Iodine-131(I131) decaying energy were taken equivalent, where I131 is the commonest safely used radionuclide [17,21]. Thus the conversion factor from Emad unit to Mega electron volt (MeV) unit is as follows [10–30]:

(2) 1Emad=23234.59MeV.

This concept for DT–EC in the biological systems was established to asses the limits of energy that is suitable for energy conversion processes.

Monitoring the mechanical behavior of the tumor response to therapy is assessed by determining the growth/or shrinkage constants of those tumors of different volumes along the corresponding periods [21–24]. The growth constant ($\frac{\mathrm{ln2}}{t_{D}}$, where $t_{D}$ is the tumor doubling time in seconds)/or shrinkage constant ($\frac{\mathrm{ln2}}{t_{1/2}}$, where $t_{1/2}$ is the tumor half-life time in seconds) of the tumor at a certain time expresses the rate of the difference between mitosis and apoptosis with respect to the total number of the tumor cells (M–A) that characterize the tumor response at that time [10–17]. If rate of mitosis is greater than that of apoptosis, tumor grows by the growth constant, and vice versa if rate of mitosis is less than that of apoptosis, tumor shrinks by the shrinkage constant [10–14].

Tumor $t_{D}$ intraday increases linearly with time for specific initial and final volumes according to the exponential growth model as follows [10–17,27–30]:

(3) $$\mathrm{Tumor}\;t_{D}\;\mathrm{intraday}=\frac{\mathrm{ln2}}{\ln V_{\mathrm{Final}}-\ln V_{\mathrm{Initial}}}\times t\;\left(s\right).\;$$

To apply Eq. (3) in the case of shrinking for tumor of volume (*V*), the apoptotic tumor portion of half-life time ${(t}_{1/2})$ would be replaced by a virtual growth portion of doubling time ($t_{D}$) equivalent to the growing portion before undergoing apoptosis as follows [10–14]:

(4) $${\left(\frac{V_{\mathrm{Initial}}-V_{\mathrm{Final}}}{V_{\mathrm{Initial}}}\right)}_{\mathrm{Shrinkage}}={\left(\frac{V_{\mathrm{Initial}}}{V_{\mathrm{Final}}-V_{\mathrm{Initial}}}\right)}_{\mathrm{Virtualgrowth}}.$$

The clinical staging model presented by Moawad showed that the tumor energy that expresses the tumor histologic grade ${(H}_{G})$ can be identified using the formula of DT–EC induced in tumor cells during tumor formation or therapy as follows:

(5) $$H_{G}=\ln{\left(\ln\frac{\mathrm{ln2}}{t_{D}}\right)}^{2}\times C_{0}\times h\times 23234.59\;\mathrm{MeV},$$

where $C_{0}\times h$is number of the hypoxic cells in the tumor or number of the inoculated cells in the transplanted tumor in xenografted models [10–17,27–30].

### 3.3 Identifying effectiveness and optimal regimens of cell cycle specific antitumor drugs

Accordingly from Eq. (5), the alteration in the treated tumor $H_{G}$ compared to that of the control tumor induced by the drug dose would be equivalent to the energy yield by the drug dose according to the following model [10–20]:

(6) $$E_{\mathrm{Dose}}=\left[\ln{{\left(\ln\left(\frac{\ln2}{t_{D}}\right)\right)}^{2}}_{\mathrm{Treated}}-\ln{{\left(\ln\left(\frac{\mathrm{ln2}}{t_{D}}\right)\right)}^{2}}_{\mathrm{Control}}\right]\times C_{0}\times h\times 23234.59\;\mathrm{MeV}.$$

Assessment of the efficient regimen for optimizing therapy would be based on achieving an accumulated doubling time–energy conversion in the tumor cells by the doses of the regimen [10–17].

For cell cycle specific antitumor drugs – as docetaxel –, the higher the energy yields by the same drug dose the more effectiveness of the applied regimen and vice versa [12].

In addition, as much as the time period (*t*) from initiating therapy passes in the optimal cell cycle specific treatments the induced tumor doubling time ${(t}_{D})$ intraday should be steadily increased.

Thus, the criterion of the efficient regimen of docetaxel treatment can be determined by comparing the tumor $t_{D}$ intraday on time of dose delivery to time periods from the start of therapy to the time of dose delivery in the studied regimen.

## 4 Experimental design, materials and methods

Monitoring the growth constant in each of the treated and control groups for tumor models was identified by applying the exponential growth model shown in Eq. (3) on the progress induced in tumor volume illustrated in Table 1. In case of shrinking, the tumor׳s shrinkage portion was replaced by the growing portion before undergoing apoptosis as determined from Eq. (4) in the exponential growth model shown in Eq. (3) to identify the virtual growth constant.

$H_{G}$ of all treated and control groups has been identified by applying DT-EC formula on their determined growth constants and knowing their numbers of the inoculated cells in the transplanted tumor of those xenografted models from Table 1 as shown in Eq. (5).

Determining the energy yield by the docetaxel dose that equivalent to the alteration in the treated tumor $H_{G}$ compared to that of the control tumor for each tumor model as shown in Eq. (6).

**Fig. 1:**
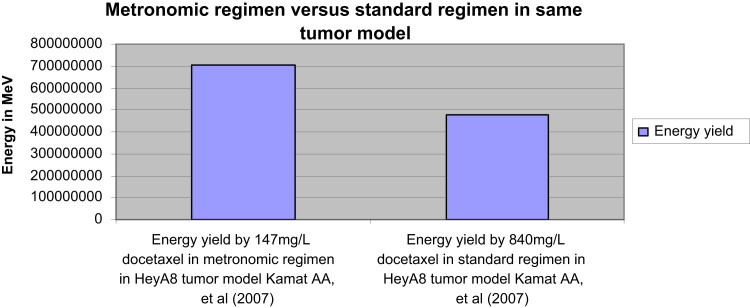


**Fig. 2:**
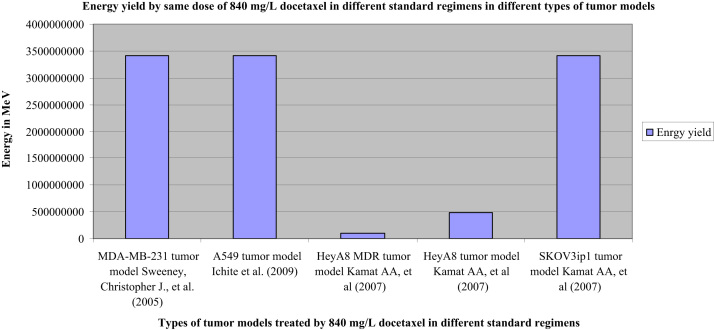


Data of the energy yield by docetaxel doses in the treated groups demonstrates puzzling response to docetaxel therapy illustrated in Fig. 1 and 2.

In same tumor model, data shows the metronomic regimens of low doses could be more efficient than the standard regimens of high doses.

Fig. 1 shows two regimens of docetaxel were applied on the same tumor model ((2.5×10^5^) HeyA8 cells). The lower dose (147 mg/L) in metronomic regimen was more effective than the higher one (840 mg/L) in standard regimen.

Although data shows that the therapeutic effect of same dose of docetaxel in different tumor models is unpredictable, data shows also that the therapeutic effect of same dose was identical in some standard regimens applied on different tumor types. Consequently, the response to those regimens of the identical therapeutic effect could be predicted.

In Fig. 2, the therapeutic effect of same dose of docetaxel (840 mg/L) in different standard regimens applied on five different tumor types was identical in three of them and different in the others.

In addition, tumor doubling time intraday on time of dose delivery of the applied regimens on tumor models shown in Table 1 has been identified using Eq. (3) to be compared by the time period from the start of therapy to the time of next dose delivery in the studied regimens.

**Fig. 3:**
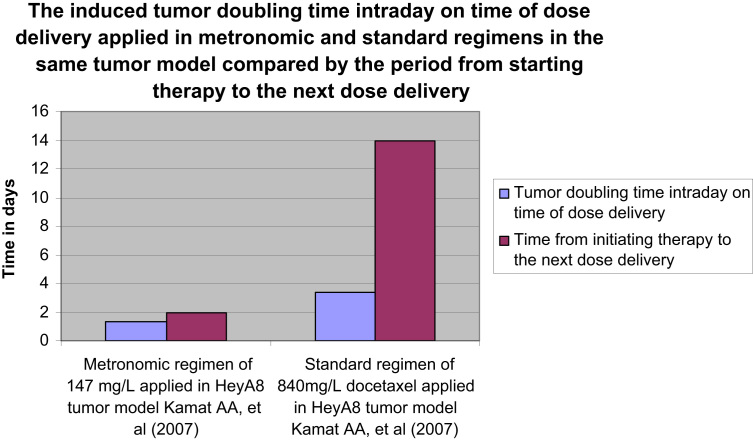


**Fig. 4:**
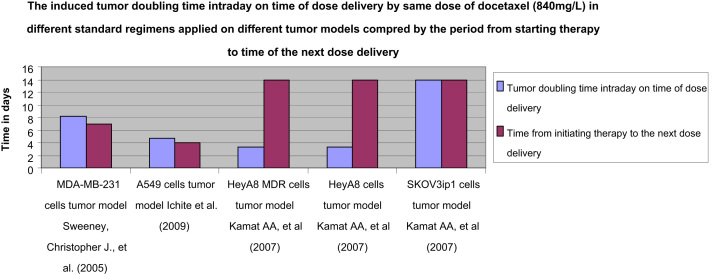


Data of the induced tumor doubling time on time of dose delivery compared by the period from starting therapy to time of the next dose delivery would clarify when the therapeutic effect of same docetaxel doses would be optimized, identical and consequently predictable for its consistency, or on the contrary when it would be puzzling and randomized and consequently unpredictable in the treated groups as illustrated in Figs. 3 and 4.

Fig. 3 shows the induced tumor doubling time intraday on time of dose delivery by different doses of docetaxel (147 mg/L, 840 mg/L) applied in metronomic and standard regimens respectively on the same tumor model ((2.5×10^5^) HeyA8 cells) compared by the time period from the start of therapy to the time of dose delivery in the studied regimen.

Fig. 4 shows the induced tumor doubling time intraday on time of dose delivery by the same dose of docetaxel (840 mg/L) applied in different standard regimens on five different tumor models compared by the time period from the start of therapy to the time of the next dose delivery in the studied regimen.

By completing analysis to the acquired data for all tumor models shown in Table 1, steps described in Section 2 can be performed and then, one can establish the optimal standard regimen and predicting the response to docetaxel therapy.

## Conflict of interest

The author declares no conflict of interest.

## Figures and Tables

**Table 1:** Data presented in several studies of the docetaxel anticancer effect on different types of tumor models of different cell lines [1].

Treatment Number	Authors	Injected cell line	Docetaxel dose $\left(\mathrm{\mu g}/\mathrm{ml}\right)$	Regimen	Control tumor volume $\left({\mathrm{cm}}^{3}\right)$	Treated tumor volume $\left({\mathrm{cm}}^{3}\right)$
1	Kamat et al. [2]	(2.5×10^5^) HeyA8 cells	147	0.5 mg/kg thrice weekly for 3.5 weeks	From 0.1 to 1.2 in 3.5 weeks	From 0.1 to 0.288 in 3.5 weeks
2	Williams et al. [3]	(1×10^6^) MAT-LyLu (MLL) cells	392	Two doses of 7 mg/kg on days 4 and 11	From 0.5 to 4.8 in 10 days	From 0.5 to 4.4 in 10 days
3	Liu et al. [4]	(5×10^6^) Hep-2 cells	420	Two doses of 7.5 mg/kg/week	From 0.15 to 0.45 in 14 days	Shrunk from 0.15 to 0.09 in 6 days and then grew from 0.09 to 0.17 in 8 days
4	Li et al. [5]	(1×10^6^) PC-3 cells	420	Three doses of 5 mg/kg on 6 days	From 0.57 to 1.93 in 11 days	From.54 to 1.28 in 11 days
5	Banerjee et al. [6]	(1×10^6^) C4-2b cells	560	5 mg/kg body weight given i.v. every 3rd day (total of four doses)	From 0.1 to 0.99 in 31.5 days (4.5 weeks)	From 0.1 to 0.371 in 31.5 days (4.5 weeks)
6	Williams et al. [3]	(1×10^6^) MAT-LyLu (MLL) cells	649.6	11.6 mg/kg on days 4 and 11	From 0.5 to 4.8 in 10 days	From 0.5 to 1.63 in 10 days
7	Sweeney et al. [7]	(1×10^6^) MDA-MB-231 cells	840	5 mg/kg/week for 6 weeks	From 0.06 to 0.24 in 14 days	From 0.232 to 0.42 in 17 days
8	Ichite et al. [8]	(1×10^6^) A549 cells	840	10 mg/kg on days 14, 18 and 22	From 0.05 to 0.26 in 14 days	From 0.05 to 0.09 in 14 days
9	Kamat et al. [2]	(1×10^6^**)** SKOV3ip1 cells	840	15 mg/kg/2 weeks for 4 weeks	From 0.1 to 0.75 in 3.5 weeks	From 0.1 to 0.2 in 3.5 weeks
10	Kamat et al. [2]	(1×10^6^**)** HeyA8 MDR cells	840	15 mg/kg/2 weeks for 4 weeks	From 0.1 to 2.2 in 3.5 weeks	From 0.1 to 2.0 in 3.5 weeks
11	Kamat et al. [2]	(2.5×10^5^) HeyA8 cells	840	15 mg/kg/2 weeks for 4 weeks	From 0.1 to 1.2 in 3.5 weeks	From 0.1 to 0.42 in 3.5 weeks
12	Yoo et al. [9]	(15×10^6^) of HNSCC line; HN30	2100	7.5 mg/kg per injection twice a week for 6 weeks	From 0.4 to 1.7 in 35 days	From 0.4 to 0.192 in 35 days
13	Yoo et al. [9]	(15×10^6^) of HNSCC lines; HN30	5040	15 mg/kg per injection twice a week for 6 weeks	From 0.4 to 1.7 in 35 days	From 0.4 to 0.02 in 85 days
14	Yoo et al. [9]	(15×10^6^) of HNSCC line; and HN12	5040	15 mg/kg per injection twice a week for 6 weeks	From 0.25 to 2.5 in 35 days	From 0.25 to 0.05 in 40 days
