# Supplementary material for: Data to establish the optimal standard regimen and predicting the response to docetaxel therapy
Source: Data Brief. 2015 Oct 9;5:439–46. doi: 10.1016/j.dib.2015.09.033 (PMC4610957; doi:10.1016/j.dib.2015.09.033)
Supplement: Supplementary file 1 — Supplementary material [file mmc1.doc]

### Conflict of interest

### The author declares no conflict of interest
